# Fabrication of CP-Ti structure with controllable wettability using powder bed fusion and eco-friendly post-process

**DOI:** 10.1038/s41598-024-54958-1

**Published:** 2024-02-26

**Authors:** Won-Jung Oh, Donghyun Kim, Dong-Hyun Kim, Doo-Man Chun, Jeonghong Ha, Chung-Soo Kim

**Affiliations:** 1 Smart Forming Process Group and 3D Printing Manufacturing Process Center, Korea Institute of Industrial Technology (KITECH), 40, Techno saneop-ro 29beon-gil, Nam-gu, Ulsan, Republic of Korea; 2 Department of Mechanical Engineering, University of Ulsan, Ulsan, Republic of Korea

**Keywords:** Powder bed fusion, Silicone oil coating, Hydrophobic surface, Wettability control, Chemical engineering, Mechanical engineering

## Abstract

Hydrophobic surfaces have a wide range of applications, such as water harvesting, self-cleaning, and anti-biofouling. However, traditional methods of achieving hydrophobicity often involve the use of toxic materials such as fluoropolymers. This study aims to create controllable wettability surfaces with a three-dimensional geometry using a laser base powder bed fusion (PBF) process with commercially pure titanium (CP-Ti) and silicone oil as non-toxic materials. The optimal PBF process parameters for fabricating micropillar structures, which are critical for obtaining the surface roughness necessary for achieving hydrophobic properties, were investigated experimentally. After fabricating the micropillar structures using PBF, their surface energy was reduced by treatment with silicone oil. Silicone oil provides a low-surface-energy coating that contributes to the water-repellent nature of hydrophobic surfaces. The wettability of the treated CP-Ti surfaces was evaluated based on the diameter of the pillars and the space between them. The structure with the optimal diameter and spacing of micropillars exhibited a high contact angle (156.15°). A pronounced petal effect (sliding angle of 25.9°) was achieved because of the morphology of the pillars, indicating the controllability of wetting. The micropillar diameter, spacing, and silicone oil played crucial roles in determining the water contact and sliding angle, which are key metrics for surface wettability.

## Introduction

**Figure 1:**
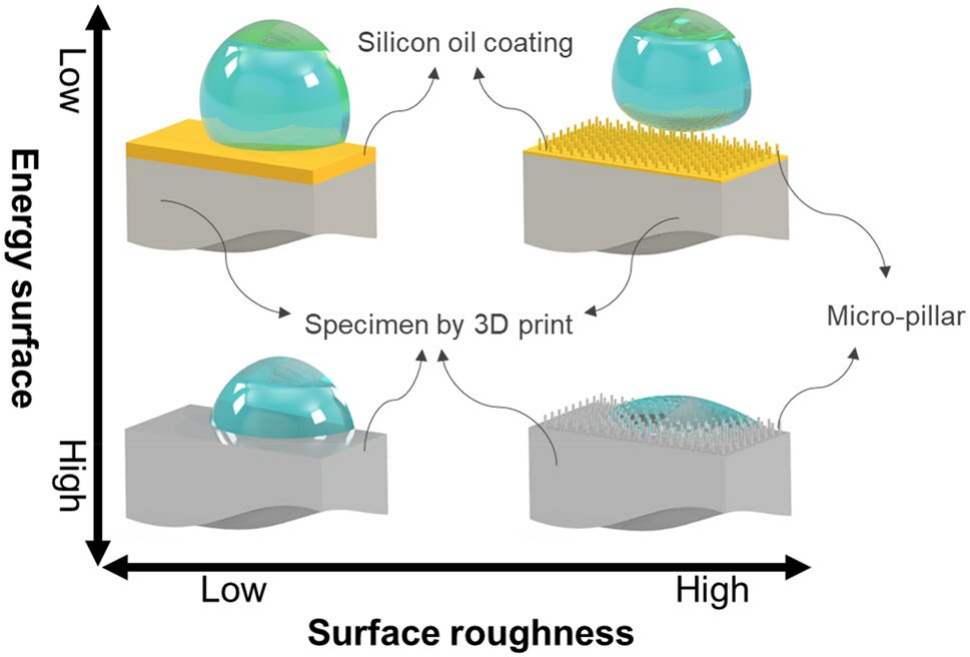
Main conditions for a surface to achieve hydrophobicity.

Hydrophobicity, often termed the lotus effect, is a phenomenon characterized by outstanding water repellency and high contact angles, typically exceeding 150°, on a surface^1–3^. Two main conditions must be satisfied for a surface to achieve hydrophobicity: low surface energy and a hierarchical nano/microscale surface structure, as shown in Fig. 1. Challenges in terms of materials and processes have been carried out to achieve two main conditions^4,5^. Despite these challenging prerequisites, hydrophobic surfaces are effective for applications such as self-cleaning^6^, drag force reduction^7^, anti-icing^8^, oil–water separation^9^, corrosion prevention^10^, noise mitigation^11^, and minimizing bacterial adhesion^12^.

Consequently, owing to their potential applications in diverse sectors, including the shipbuilding/offshore and aerospace industries, hydrophobic surfaces have garnered substantial interest^13,14^. In specific study cases for an industry where hydrophobic surfaces are applied, Wu et al. applied the nanostructured surface of the film to increase hydrophobicity and anti-reflectivity, thereby improving the stability and efficiency of energy harvesting in solar thermal power generation^15^. Alimohammadian et al. verified the oil–water separation effect by controlling the surface hydrophobicity according to the strength of the external magnetic field using a composite of graphene, iron, and iron oxide^16^. Xie et al. confirmed the possibility of freshwater harvesting using the superhydrophobic and photothermal properties of polyethylene/carbon nanotube foam^17^.

Conventional techniques for fabricating hydrophobic surfaces include dip-coating, chemical bath deposition, electrostatic layer-by-layer assembly, surface etching, and chemical vapor deposition^18^. In addition, laser-based processes, such as laser marking, laser ablation, and laser interference lithography, have been recognized for their proficiency in generating large-area patterns within the submicron to micron scale, thereby effectively crafting superhydrophobic surfaces^14^. Therefore, continuous efforts have been made to achieve superhydrophobic characteristics by utilizing a laser to texture various metals such as copper, stainless steel, and titanium^19–24^.

Although these methods often entail complex and intricate procedures leading to elevated production costs and limited scalability, recent advancements in 3D printing technology offer a compelling solution to these challenges. This opens the door to broader applications of hydrophobicity in various industrial sectors. By leveraging additive manufacturing, it is feasible to design detailed nano/microscale structures to ensure controlled surface roughness and morphology, both of which are pivotal for inducing hydrophobic characteristics. Numerous examples demonstrate the successful incorporation of 3D printing into the crafting of hydrophobic surfaces^25,26^. In this context, the term '3D printing' refers to binder jetting (BJT), directed energy deposition, material extrusion, material jetting, powder bed fusion (PBF), sheet lamination, and vat photopolymerization, as defined in ASTM 52900.

With non-metallic materials such as polymers, a higher resolution can be achieved compared with that of metal 3D printing. This is largely because these processes employ relatively low-energy sources rather than high-energy sources, such as lasers or electron beams. Instances of 3D printing that leverage these advantages, creating superhydrophobic surfaces with micro-hole structures of 100 µm or less, or incorporating nano-sized porosity, have been documented^27,28^.

Despite challenges such as relatively low resolution and high surface energy, efforts have been made to attain controllable wettability in metal 3D printing surface structures. In particular, researchers in the field of metal 3D printing have employed various strategies using powder bed base 3D printing such as metal BJT and PBF. The principle of powder bed base 3D printing is to selectively crosslink or fuse the powder bed to fabricate a 3D object. The process begins with the slicing of 3D objects into layer thicknesses. Each sliced layer image is then deposited onto a build platform. The binder or laser is then used to selectively crosslink or fuse the powder in the desired region. The build platform is then lowered by layer thickness, and a new layer of powder is spread on the previous layer. These processes are repeated until the entire object is complete. A number of structures for controllable wettability surfaces have been fabricated by powder bed base 3D printing. Some studies include: circular^29^, protruding trapezoidal^30^, and inverted trapezoidal micropatterns^31^. In these studies, metal powders, including AISI 316 L, copper, and titanium, were used. Additionally, a non-traditional 3D printing technique involves micromesh grid texturing with copper, which is achieved through selective laser melting of the ink-printed process^32^. Moreover, one study highlighted the modulation of the water contact angle on CP-Ti components produced via PBF, which was achieved through an anodization process^33^. Despite the promising benefits of these surfaces, traditional methods for achieving the hydrophobicity of metallic surfaces often involve the use of toxic materials such as fluoropolymers, raising concerns about their environmental impact and long-term sustainability^31^.

In response to these concerns, this study aims to develop an eco-friendly alternative to conventional hydrophobic surfaces by employing a laser-based PBF process with commercially pure titanium (CP-Ti) and silicone oil as nontoxic materials. This study focuses on creating hydrophobic surfaces with three-dimensional geometries, which are essential for various practical applications.

A critical aspect of this research involves investigating the optimal PBF process parameters for fabricating micropillar structures, which are necessary to obtain the surface roughness required to achieve hydrophobic properties. By adopting an experimental approach, this study aims to identify the most suitable parameters for producing the desired surface features while maintaining the structural integrity and functionality of the fabricated CP-Ti components.

Upon successful fabrication of micropillar structures through PBF, the surface energy of the resulting structures was reduced by treatment with silicone oil. As an eco-friendly and non-toxic alternative to fluoropolymers, silicone oil provides a low-surface-energy coating that contributes to the water-repellent nature of hydrophobic surfaces^34^.

The wettability of the treated CP-Ti surfaces was evaluated based on the diameters of the micropillars and the pitch between them. By identifying the optimal micropillar diameter and pitch, this study successfully achieved a water contact angle of 156.15°, indicating hydrophobic behavior. The results demonstrate that the micropillar diameter, pitch, and silicone oil treatment play crucial roles in determining the water contact angle, which is a key metric for assessing the hydrophobicity of a surface.

## Experimental

### Powder bed fusion and post-processing

**Figure 2:**
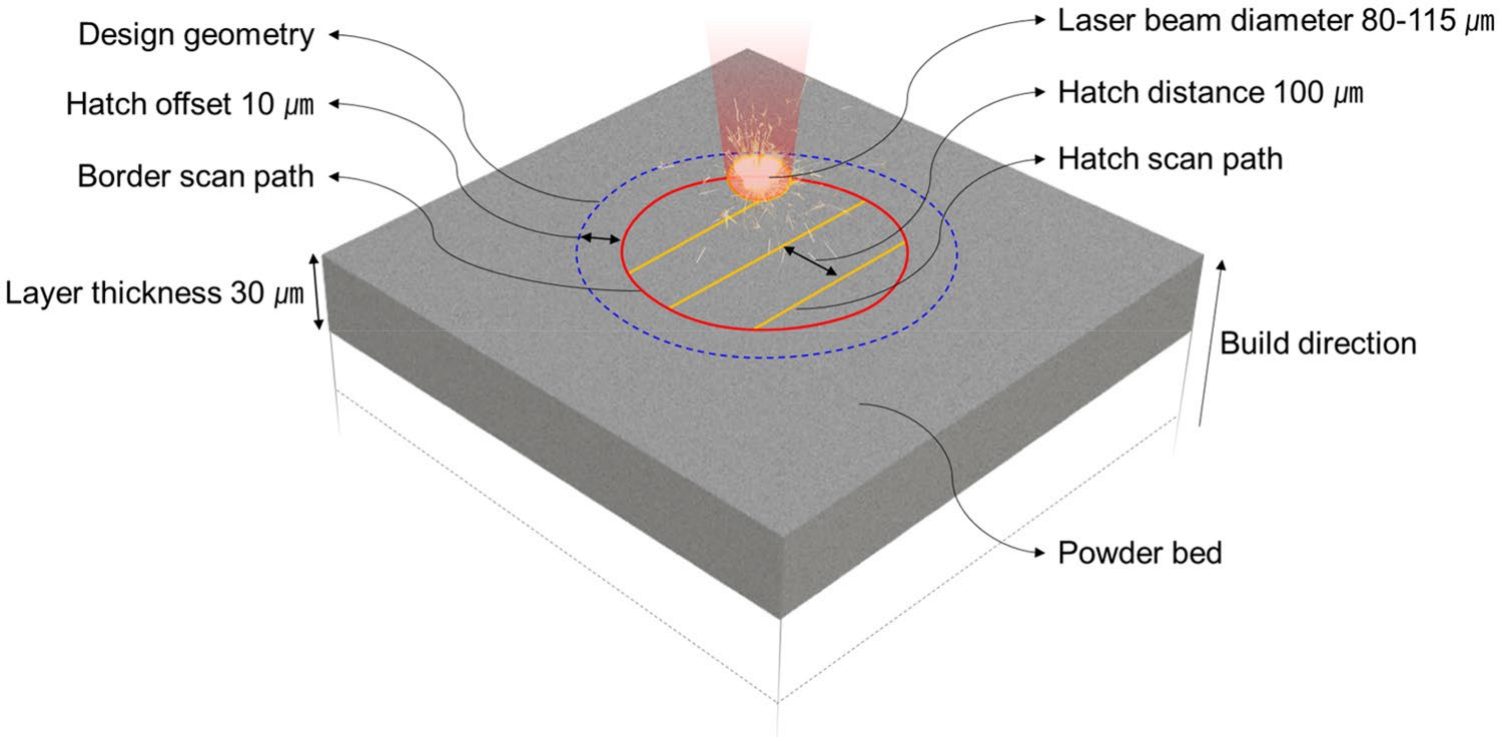
Laser scan strategy in PBF.

**Figure 3:**
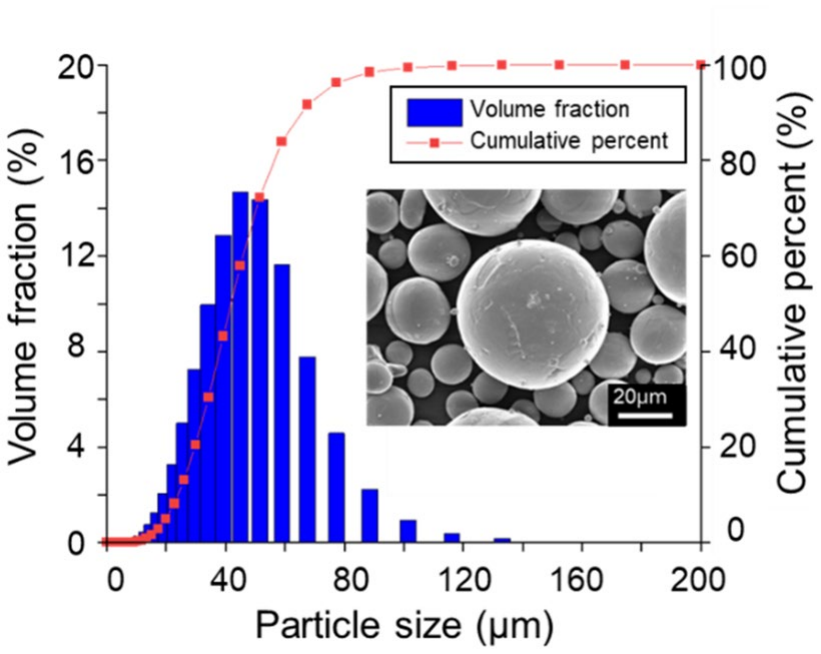
Particle size distribution of CP-Ti (Grade 2) powder, with D10: 24.0 μm, D50: 41.7 μm, and D90: 65 μm. The inset shows an SEM image of Ti Grade 2 powder.

Experiments using PBF were conducted using the SLM 280 system from SLM Solutions Group AG. The equipment features a 400 W fiber laser with a wavelength of 1064 nm, a continuous wave laser type, maximum scanning speed 4.0 m/s, and a beam focus diameter of 80–115 μm and is capable of fabricating structures with layer thicknesses ranging from 20 to 75 μm. In this study, as shown in Fig. 2, the hatch distance and layer thickness were fixed at 0.1 and 0.03 mm, respectively, whereas other key variables, such as laser power and volumetric energy density, were adjusted during the specimen fabrication process. The layer thickness was determined by powder thickness adjusted by the length the build platform descends. The volumetric energy density is defined by Eq. (1). The scan speed determined using this equation was used as the input value for the PBF device. Pure titanium (Grade 2) powder (SLM Solutions Group AG), also known as CP-Ti, was used as the deposition material, with compositions of Fe 0.3, O 0.25, C 0.08, N 0.03, and H 0.015 wt%, and balanced Ti. Melting point of CP-Ti is 1665 °C. The base plate was composed of CP-Ti. The particle size distributions of the powders are shown in Fig. 3.

1 $$Volumetric\;energy\;density = \frac{Laser\;power}{{Hatch\;distance \times Scan\;speed \times Layer\;thickness}}$$

**Figure 4:**
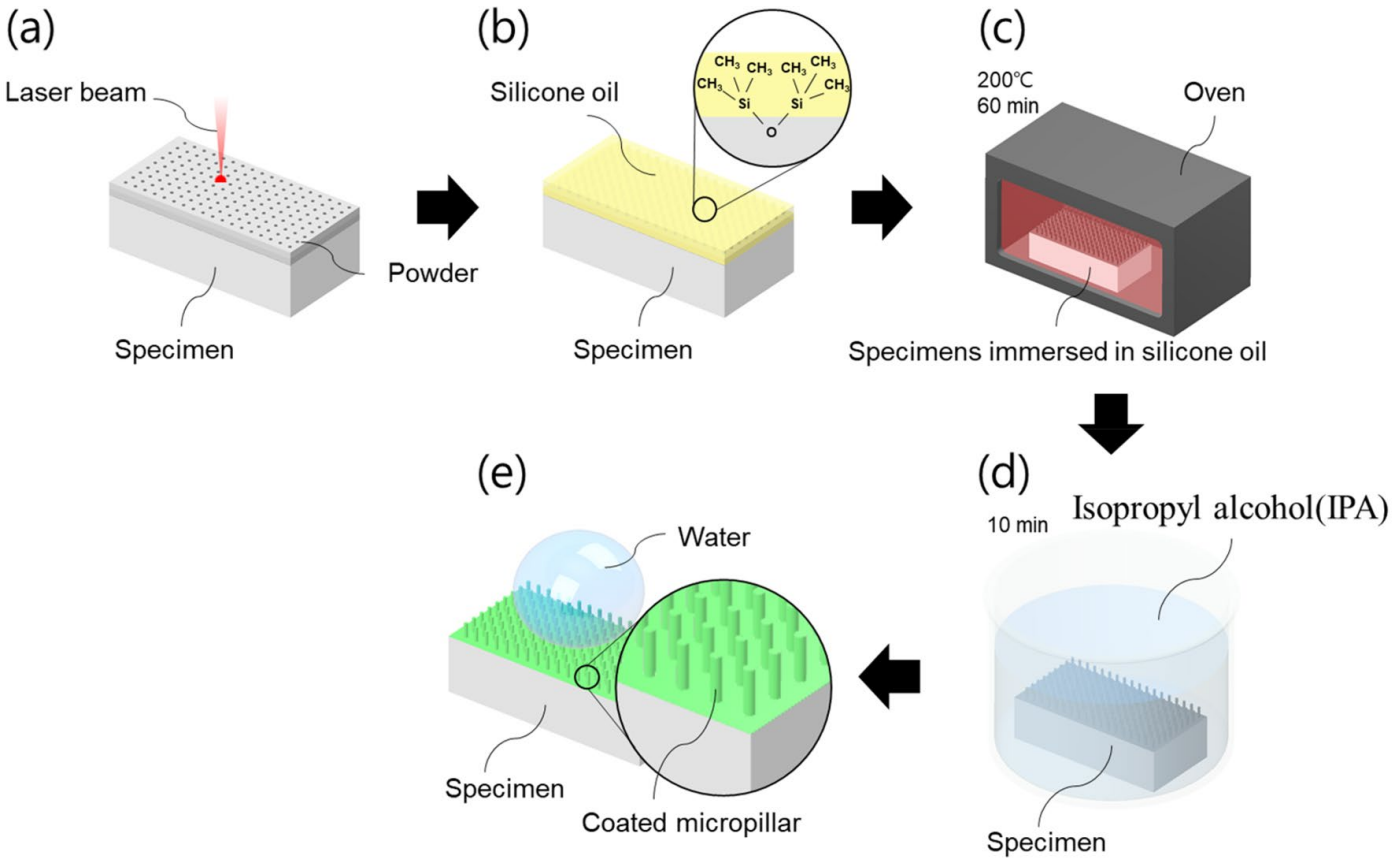
Workflow for fabricating a controllable wettability surface: (**a**) fabrication of micropillars by PBF, (**b**) dipping process, (**c**) heat treatment, (**d**) ultrasonic cleaning, and (**e**) final product.

The postprocessing procedure aimed at reducing the surface energy of the specimens fabricated via PBF, which is a pivotal aspect for attaining hydrophobicity, and is outlined in Fig. 4. Initially, the specimens were crafted using an SLM 280 system, following the prescribed PBF conditions. Once fabricated, the specimens were subjected to a dipping process submerged in silicone oil. For the chemical treatment, specimens were heat-treated in an oven at 200 °C for 60 min while immersed in the silicone oil.

This thermal step was instrumental in enhancing the adherence of the silicone oil coat to the specimen surface. Following the heat treatment, the specimens were dried and subjected to ultrasonic cleaning for 10 min in isopropyl alcohol (IPA). This cleaning regimen was deployed to eliminate any lingering contaminants and ensure an optimal bond between the silicone oil and the surface. These measures considerably reduced the surface energy. These characteristics arise from the synergy between the silicone oil coating and the surface microstructure created by the PBF technique.

### Experiment for optimization of deposition conditions

To identify the optimal deposition conditions for high relative density and low porosity, this study focused on controlling the laser power and volumetric energy density among the PBF process parameters. The laser power ranged from 85 to 160 W, whereas the volumetric energy density was set between 95 and 125 J/mm^3^, as presented in Table 1. A 10 × 10 × 10 mm cube was fabricated to measure the relative density and porosity. The relative density was determined using the Archimedes method, which provides accurate measurements of the volume and mass of an object. To assess the porosity, cross-sectional images of the specimens were obtained using an optical microscope, and the ratio between the area with pores and the normal area was calculated.

**Table 1:** Process parameters and value range of PBF.

Parameter	Value
Laser power (W)	85.0, 103.8, 122.5, 141.3, 160.0
Volumetric energy density (J/mm^3^)	95.0, 102.5, 110.0, 117.5, 125.0

### Experiment to determine micropillars for hydrophobicity

**Figure 5:**
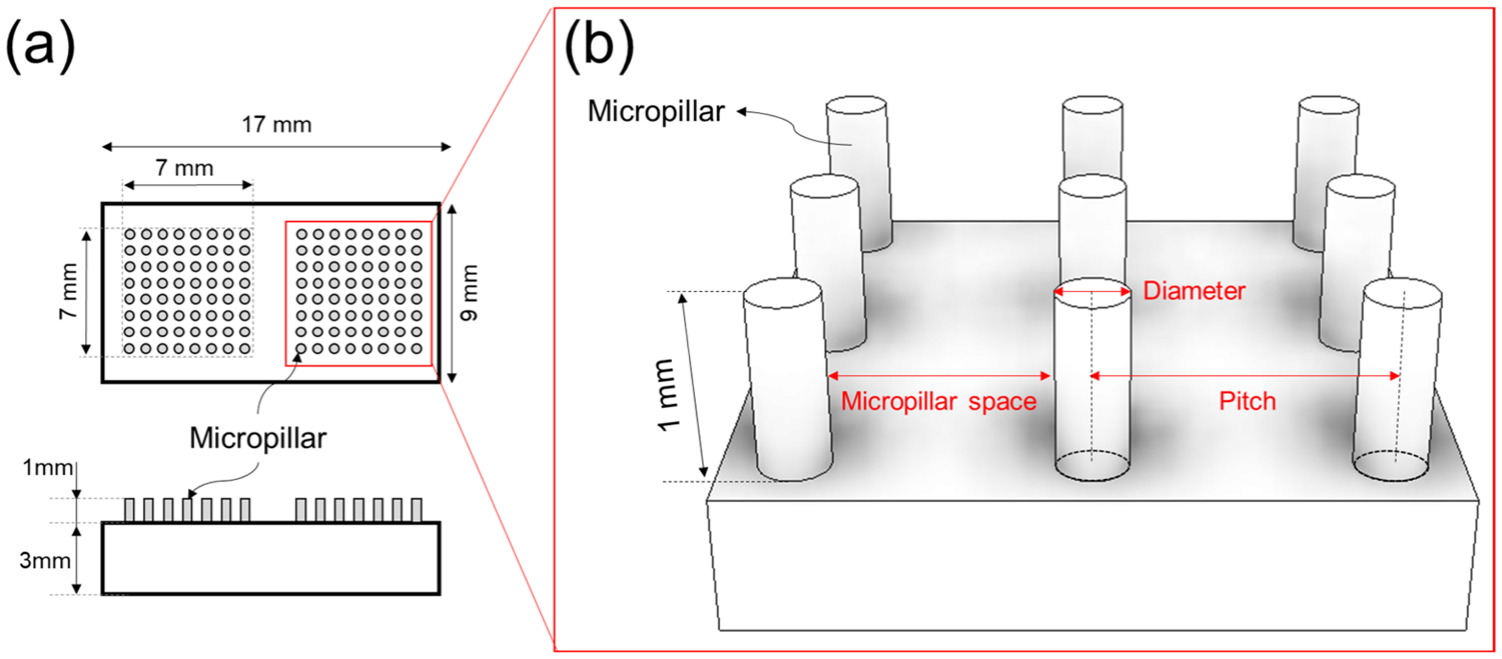
Designed micropillar: (**a**) layout diagram of micropillars, (**b**) parameters of micropillar structure.

To evaluate the hydrophobic properties of the fabricated specimens, micropillars were incorporated into the design, as shown in Fig. 5. The PBF process conditions, including a laser power of 85 W and a volumetric energy density of 125 J/mm^3^, were selected based on the relative densities and porosity values. Two sets of micropillars, each with a height of 1 mm, were placed on a 17 × 9 × 3 mm specimen. Hydrophobicity was evaluated according to the diameter of the micropillars (*D*) and the space between them (*S*). Space (*S*) means the shortest distance between the outer diameters of the pillars, and pitch (*P*) is the shortest distance between the centerlines of the pillars.

To evaluate the impact of these variables, the diameter ‘D’ ranged between 200 and 500 µm, and the spacing ‘S’ was adjusted from 100 to 500 µm, as detailed in Table 2. By methodically modulating ‘D’ and ‘S’, this experiment attempted to pinpoint the parameters that yield the maximum hydrophobicity.

**Table 2:** Diameter and pitch of micropillar.

Parameter	Value (µm)
Diameter (D)	200, 300, 400, 500
Micropillar space (S)	100, 200, 300, 400, 500
Pitch (P)	D + S

## Result and discussion

### Relative density and porosity

This study examined the effects of modulating the laser power and volumetric energy density on the relative density and porosity of CP-Ti specimens fabricated using PBF. The objective was to ascertain the prime conditions that would yield the highest relative density and the lowest porosity.

**Figure 6:**
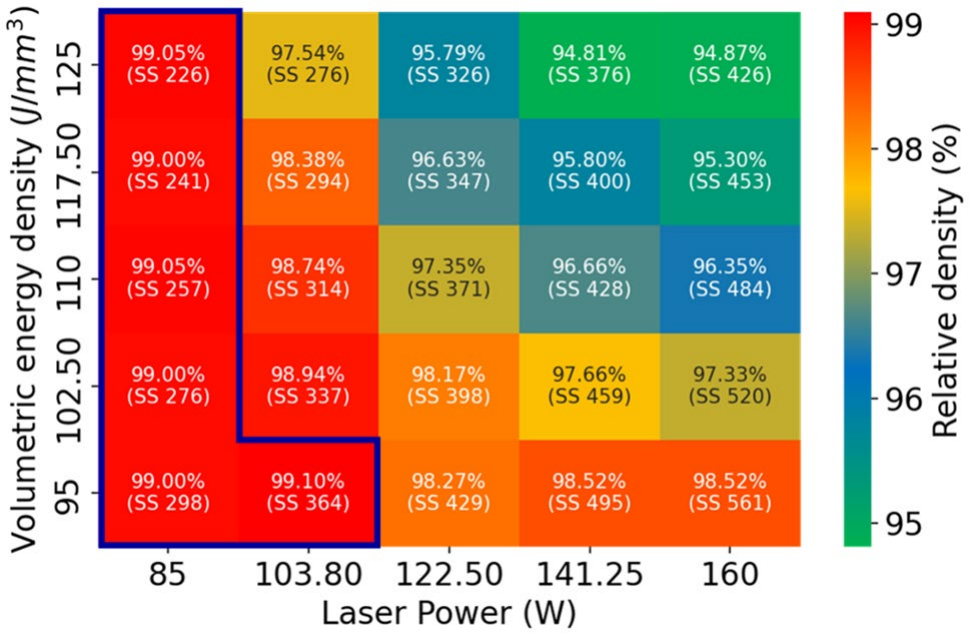
Relative density (and scanning speed) of the fabricated CP-Ti specimen according to laser power and volumetric energy density.

**Figure 7:**
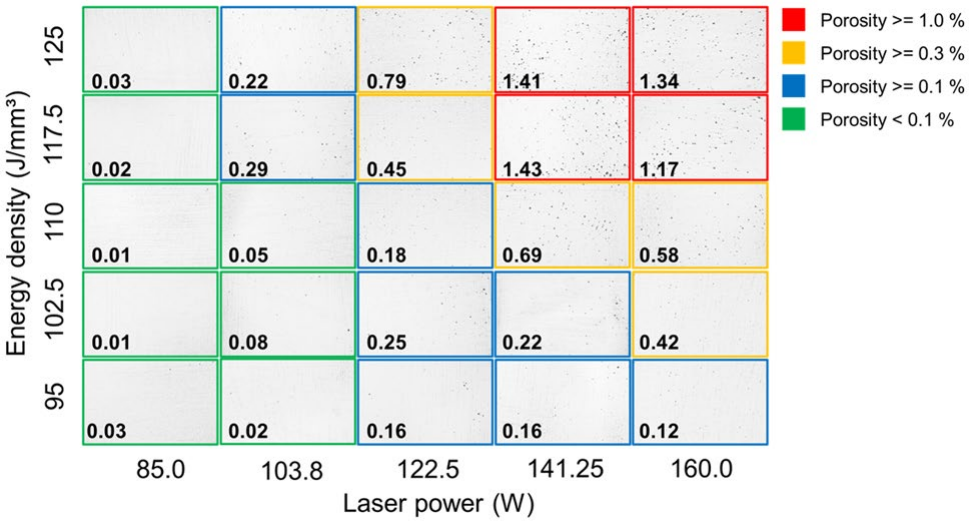
Cross-sectional microscopic image and porosity rate of the fabricated CP-Ti specimen according to laser power and volumetric energy density.

A relative density of 99% or greater was attained under six distinct conditions, as shown in Fig. 6: a laser power of 85 W with volumetric energy densities of 95, 102.5, 110, 117.5, and 125 J/mm^3^, and a laser power of 103.8 W paired with a volumetric energy density of 95 J/mm^3^. A minimal porosity rate, wherein the porous area constituted less than 0.1% of the total area, was observed under eight conditions. These are illustrated in Fig. 7: a laser power of 85 W with volumetric energy densities of 95, 102.5, 110, 117.5, and 125 J/mm^3^, alongside a laser power of 103.8 W with volumetric energy densities of 95, 102.5, and 110 J/mm^3^.

Based on our investigation, the laser power was identified as a significant factor affecting the relative density and porosity. The optimal conditions for achieving the desired relative density and porosity were determined to be a scan speed of 300 mm/s.

### Accuracy of the micropillar diameter

The accuracy of the micropillar diameter for the fabricated structures created using PBF under the same conditions as those described in “Relative density and porosity” section was evaluated. Given the challenging nature of fabricating 200 µm micropillars in a PBF system with a beam diameter of 80–115 µm, the effect of varying laser power and volumetric energy density on the precision of the micropillar diameter was investigated.

**Figure 8:**
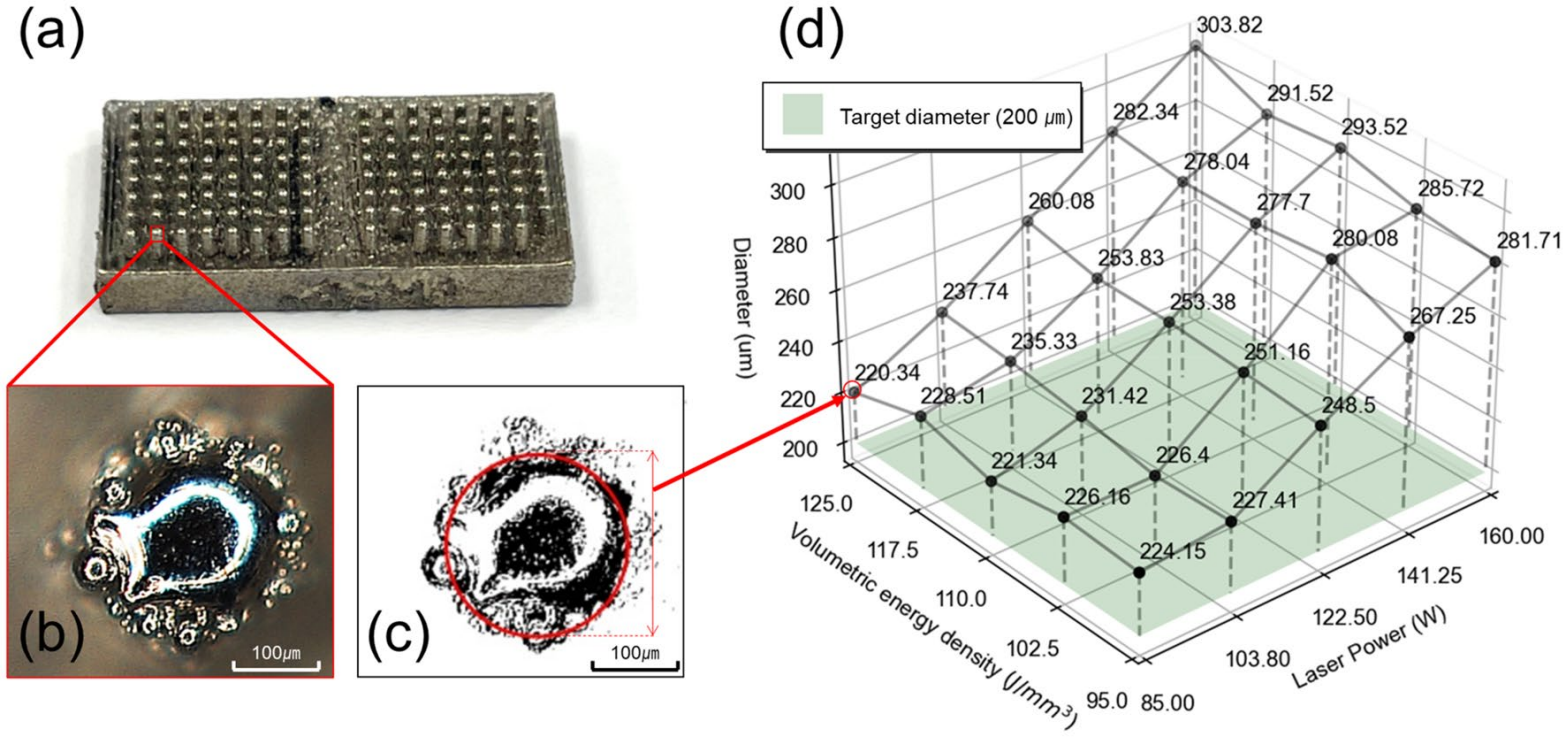
Diameter of micropillar depending on laser power and volumetric energy density (**a**) Specimen fabricated by PBF; (**b**) Image of micropillar taken with an optical microscope; (**c**) Image binarization and diameter determination of micropillars; (**d**) micropillar diameter measurement results for each parameter.

The diameters of the micropillars fabricated on the vertical surface of the sample were measured using an optical microscope, as shown in Fig. 8a and b. The images were extracted, and the pillar and non-pillar parts were separated through image binarization, as shown in Fig. 8c. The Hough transform algorithm in the OpenCV library was used to find circles based on points designated as pillars, and the diameters of the micropillars were determined accordingly. The experimental results are shown in Fig. 8d. As the laser power increased, the size of the molten pool increased, leading to a larger micropillar diameter. At the same laser power, the effect of volumetric energy density (i.e., scan speed) on the micropillar diameter was relatively small. The condition closest to the design value (200 µm), with a micropillar diameter of 220.34 µm, was achieved using a laser power of 85 W and a volumetric energy density of 125 J/mm^3^.

### Morphology of micropillars

**Figure 9:**
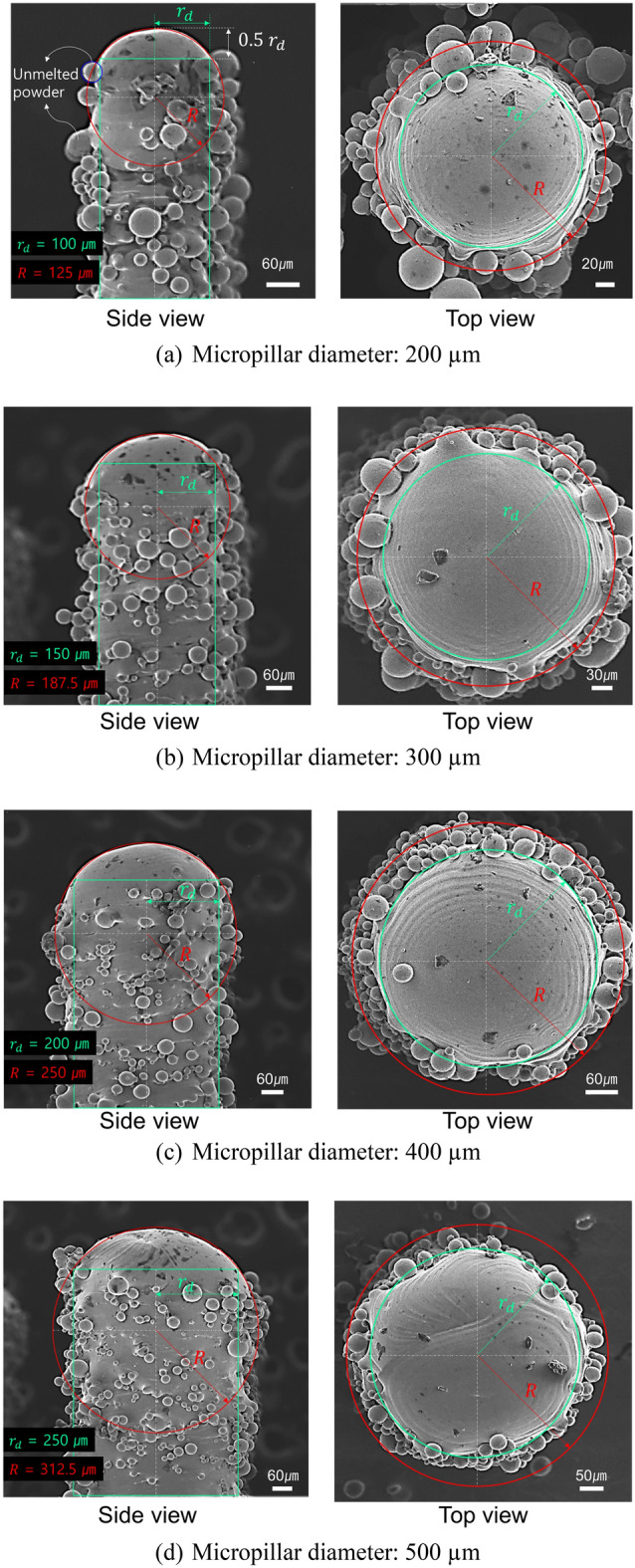
SEM image of micropillars according to the diameter.

Figure 9 shows a scanning electron microscopy (SEM) image of a micropillar designed with a cylindrical geometry and fabricated using PBF. The top surface of the micropillars appeared spherical regardless of the set diameter, whereas the side surface showed attached unmelted powder. The height of the smooth surface (sphere structure) was half the set radius of the micropillar ($r_{d}$), and the sphere structure radius was 5/4 $r_{d}$ (R). In addition, in most specimens, unmelted powder was attached beneath the smooth surface. It is interesting to note that the morphology of the micropillars was determined by certain rules for all the set diameters.

**Figure 10:**
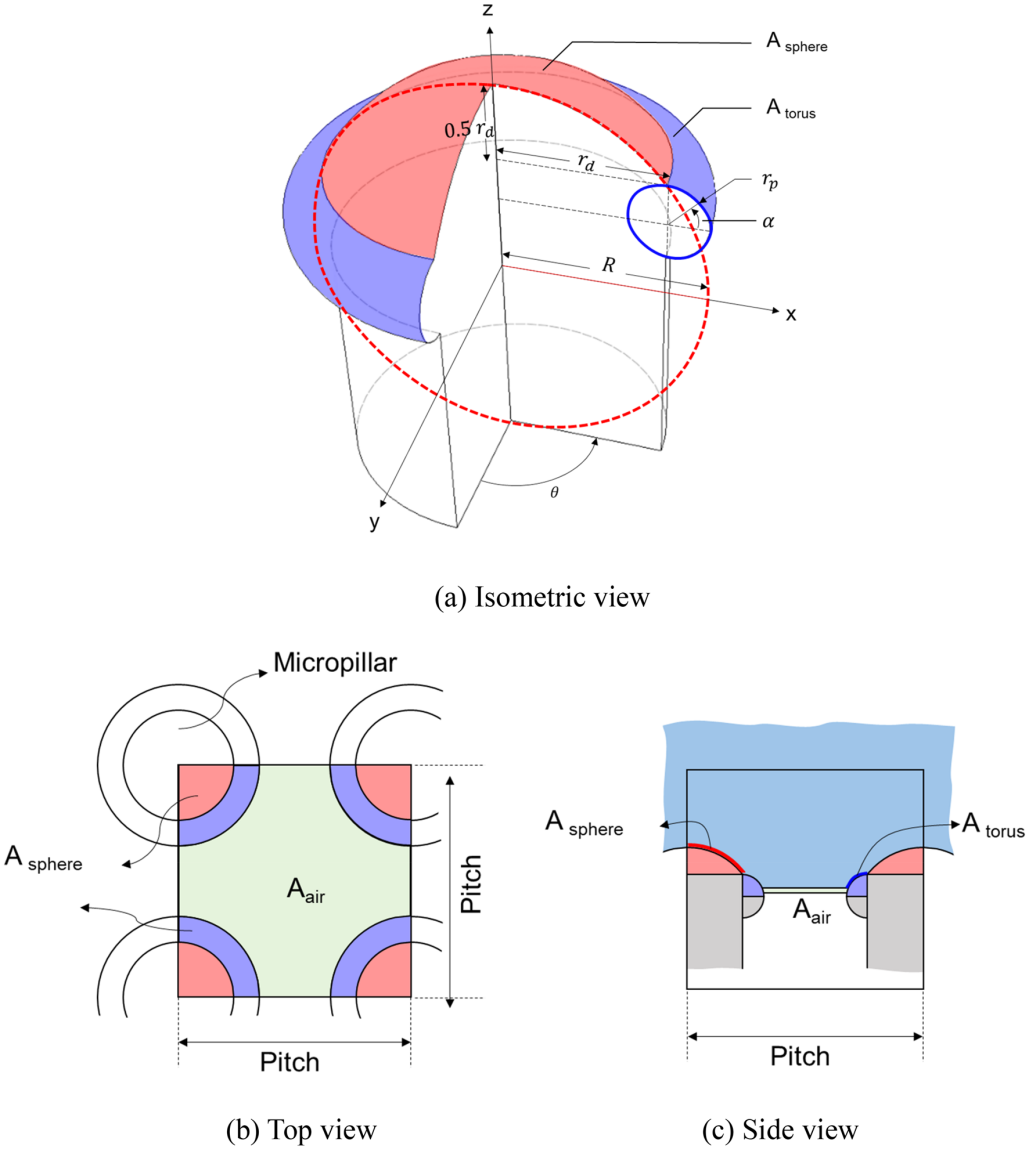
Simplified model of micropillar morphology.

To apply the Cassie–Baxter model, which is a conventional approach for determining the contact angle, it is necessary to obtain the contact area of the droplet. However, accurately defining this area is challenging because of the irregular attachment of unmelted powder. Therefore, this study proposes a simplified model, as shown in Fig. 10, assuming a torus structure for the region where the unmelted powder is present.

According to Milne^35^, the Cassie–Baxter equation that considers non-flat structures is shown in Eq. (2).

2 $$cos\theta_{c} = f_{1} cos\theta_{1} - f_{2}$$

where $\theta_{c}$ is the predicted Cassie–Baxter contact angle; $f_{1}$ and $f_{2}$ are the total area of the solid and air under the drop per unit projected area under the drop, respectively; and $\theta_{1}$ is the contact angle on the flat surface of the material.

The total area of the solid under the drop in the simplified model depends on three cases:

(i) Drop contact on the sphere surface.
(ii) Drop contact on the torus surface.
(iii) Full penetration (under the torus).

When the permeation amount is *n*, the total area of the solid under the drop in case (i) ($A_{sphere}$) can be calculated using Eq. (3).

3 $$\begin{aligned} & \left( {R - n < 0.6R} \right), \\ & A_{sphere} = \mathop \int \limits_{R - n}^{R} 2\pi \sqrt {R^{2} - z^{2} } \sqrt {1 + \left( {\frac{ - z}{{\sqrt {R^{2} - z^{2} } }}} \right)^{2} } dz \\ \end{aligned}$$

where R is $$\frac{5}{4}r_{d}$$.

The total area of the solid under the drop in case (ii) ($A_{torus}$) can be calculated using Eq. (4).

4 $$\begin{aligned} & \left( {R - n > 0.6R} \right), \\ & A_{torus} = 0.8\pi R^{2} + r_{p} \mathop \int \limits_{0}^{2\pi } d\theta \mathop \int \limits_{\beta }^{\pi /2} \left( {r_{d} + r_{p} \cos \left( \alpha \right)} \right) d\alpha \\ \end{aligned}$$

where $$\beta$$ is $$\sin^{ - 1} \left( {\frac{{r_{p} - \left( {n - 0.6R} \right)}}{{r_{p} }}} \right)$$.

### Wettability depending on the micropillar diameter and pitch

The effects of micropillar diameter and pitch on the wettability of the fabricated structures were investigated. To ensure high relative density, low porosity, and high precision in micropillar fabrication, the optimal PBF conditions were determined as follows: a laser power of 85 W, a scan speed of 226 mm/s, and a volumetric energy density of 125 J/mm^3^. A total of 20 specimens were fabricated with micropillar diameters of 200, 300, 400, and 500 µm, and micropillar spacing of 100, 200, 300, 400, and 500 µm. Because of the low laser power, unmelted powder was adhered to the surface of the micropillars, which contributed to the surface roughness required for wettability. The fabricated micropillars were post-processed using silicone oil to reduce the surface energy. The contact angles of the treated specimens, determined by placing a 10 μL water droplet on their surface, were measured to evaluate their wettability.

**Figure 11:**
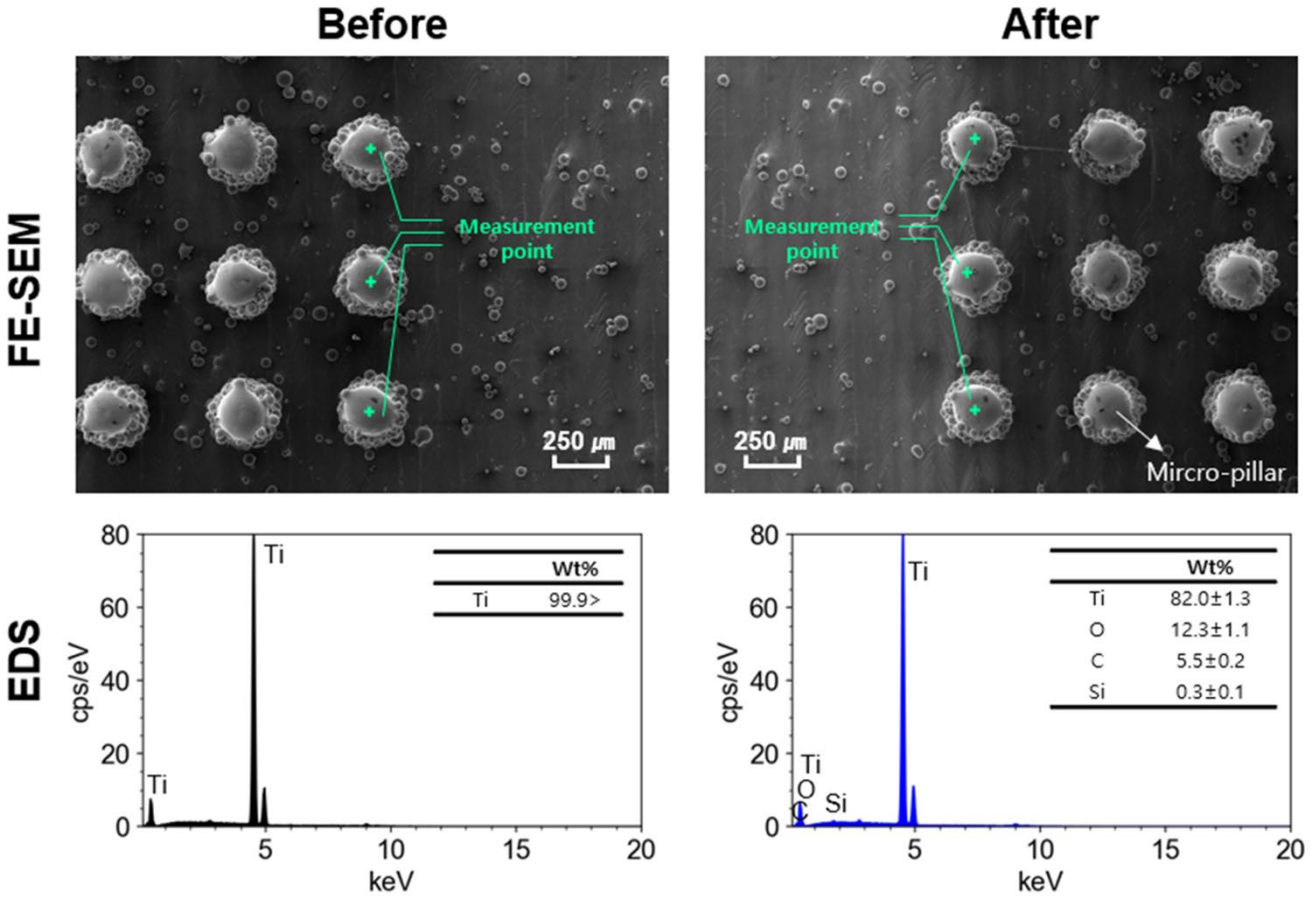
EDS analysis of surface chemical composition before and after silicone oil treatment.

Energy dispersive spectrometer (EDS) analysis revealed distinct changes in the surface chemistry following silicone oil treatment, effectively verifying its implementation as shown in Fig. 11.

**Figure 12:**
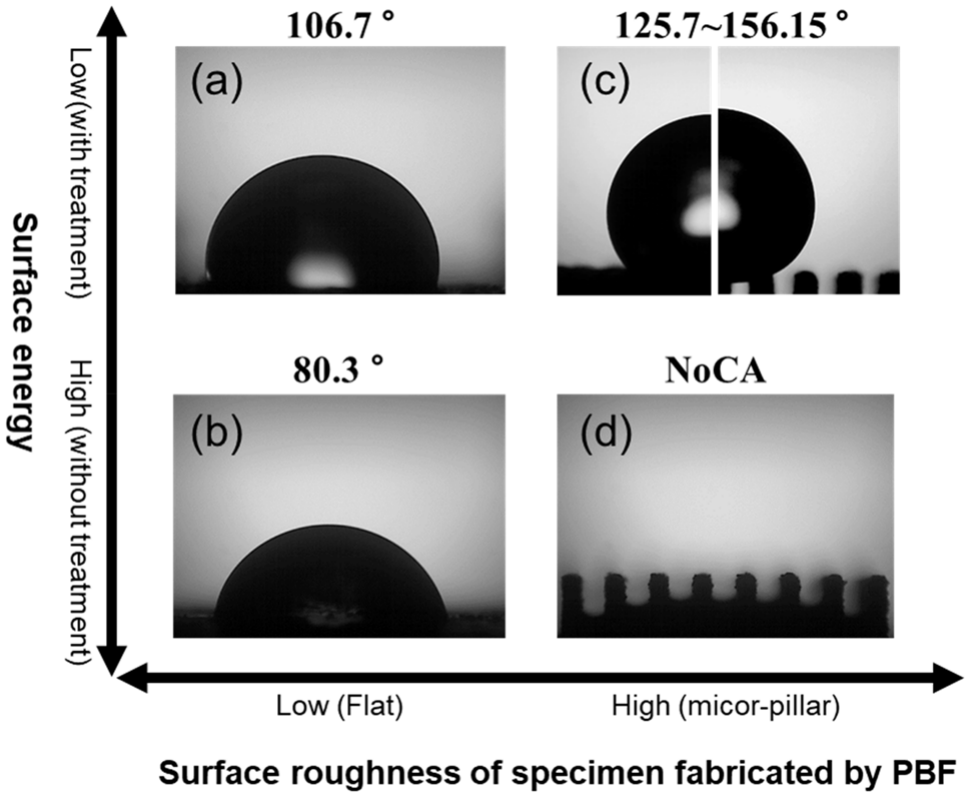
Droplet morphology on different surfaces: (**a**) on a flat surface fabricated by PBF + silicon oil treatment, (**b**) on a flat surface fabricated by PBF, (**c**) on the surface with the micro-pillar structure + silicon oil treatment, (**d**) on the surface with a the micro-pillar structure.

The experimental results for the overall contact angle are shown in Fig. 12. The contact angle varied with and without silicone oil treatment in the flat bottom surface fabricated by PBF. The contact angle of the bottom treated with silicone oil was 106.7°, and the contact angle of the floor not treated with silicone oil was 80.3°, as shown in Fig. 12a and b. The contact angles for the micro-pillar fabricated by PBF are shown in Fig. 12c and d. The untreated micro-pillar structure was hydrophilic, and no contact angle was measured. The treated micro-pillar structures showed contact angles ranging from 125.7° to 156.15°.

**Figure 13:**
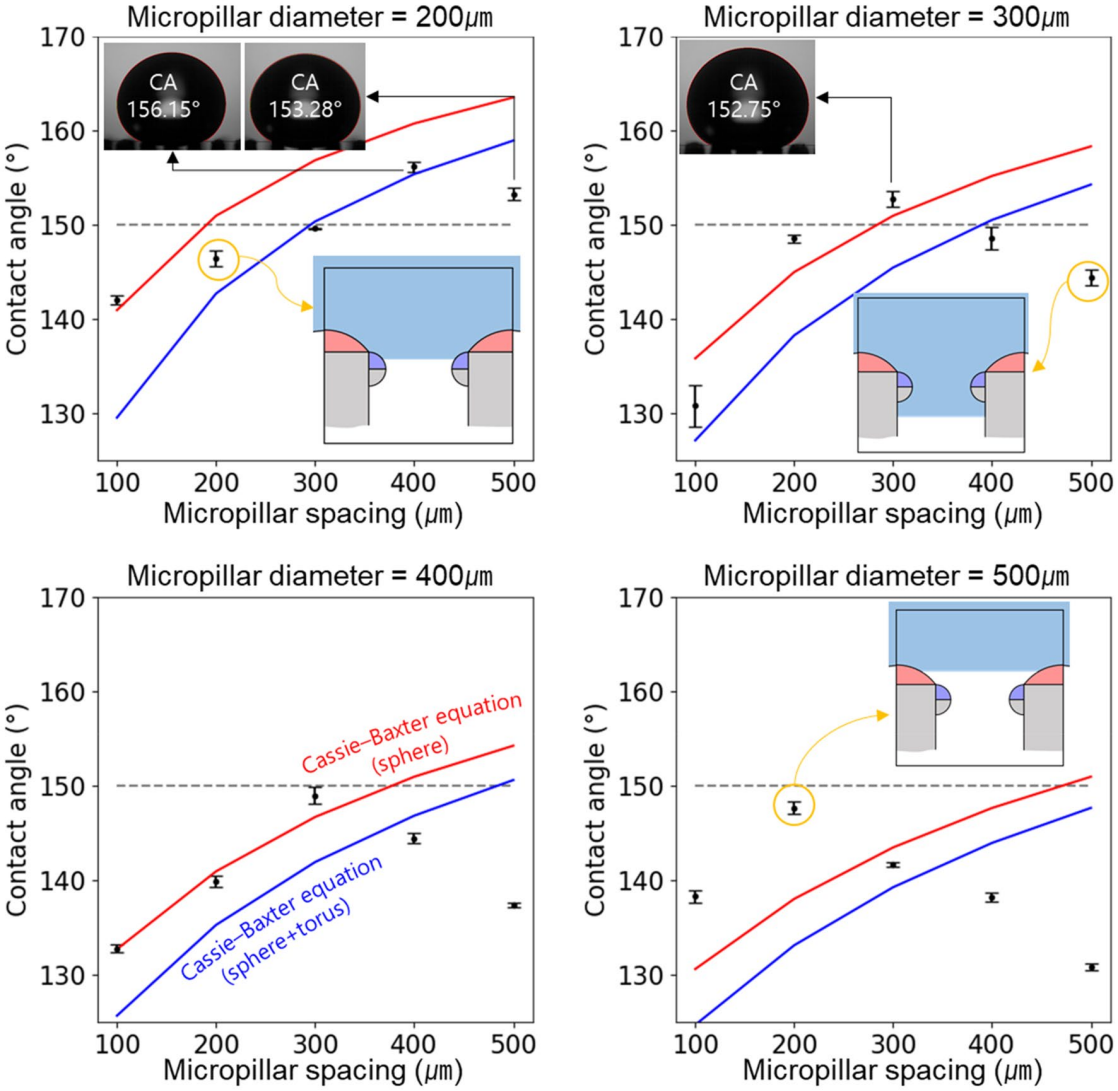
Contact angle according to diameter and spacing of micropillars.

The experimental results of the contact angle according to the diameter and spacing of the silicone oil-treated micro-pillars are shown in Fig. 13. The wider the spacing between the micro-pillars, the more liquid–air space is secured. This facilitates trapping more air between the micro-pillars, making it easier to form a Cassie–Baxter state. It is a reason that the contact angle increases as the micro-pillar spacing increases. However, a Cassie–Baxter state is metastable. If the micro-pillar spacing exceeds a certain threshold, the capillary bridge collapses, leading to the transition to the Wenzel wetting state. In these experiments, while a perfect Wenzel state wasn't observed consistently, a mixed-wetting state between Wenzel and Cassie–Baxter was found in specimens where the contact angle initially increased with increasing micro-pillar spacing and then decreased again. Low wettability, with contact angles exceeding 150°, was observed under three specific conditions: micropillar diameter of 200 µm with micropillar spacing of 400 and 500 µm, and micropillar diameter of 300 µm with micropillar spacing of 300 µm. Therefore, the proposed fabrication process effectively controlled wettability by attaining contact angles of 150° or more.

Additionally, the Cassie–Baxter model considering the spherical area (red line) and spherical + torus area (blue line), as indicated in Fig. 13, allowed the assessment of the water droplet penetration depth.

Cassie–Baxter model considering the spherical area (red line) can be expressed as the equation below.

$$cos\theta_{c} = \frac{{\max .A_{sphere} }}{{P^{2} }}cos\theta_{1} - \frac{{P^{2} - \pi \left( {r_{d} } \right)^{2} }}{{P^{2} }}$$

Cassie–Baxter model considering the spherical + torus area (blue line) can be also expressed as the equation below.

$$cos\theta_{c} = \frac{{\left( {\max .A_{sphere} + \max .A_{torus} } \right)}}{{P^{2} }}cos\theta_{1} - \frac{{P^{2} - \pi \left( {r_{d} + r_{p} } \right)^{2} }}{{P^{2} }}$$

**Figure 14:**
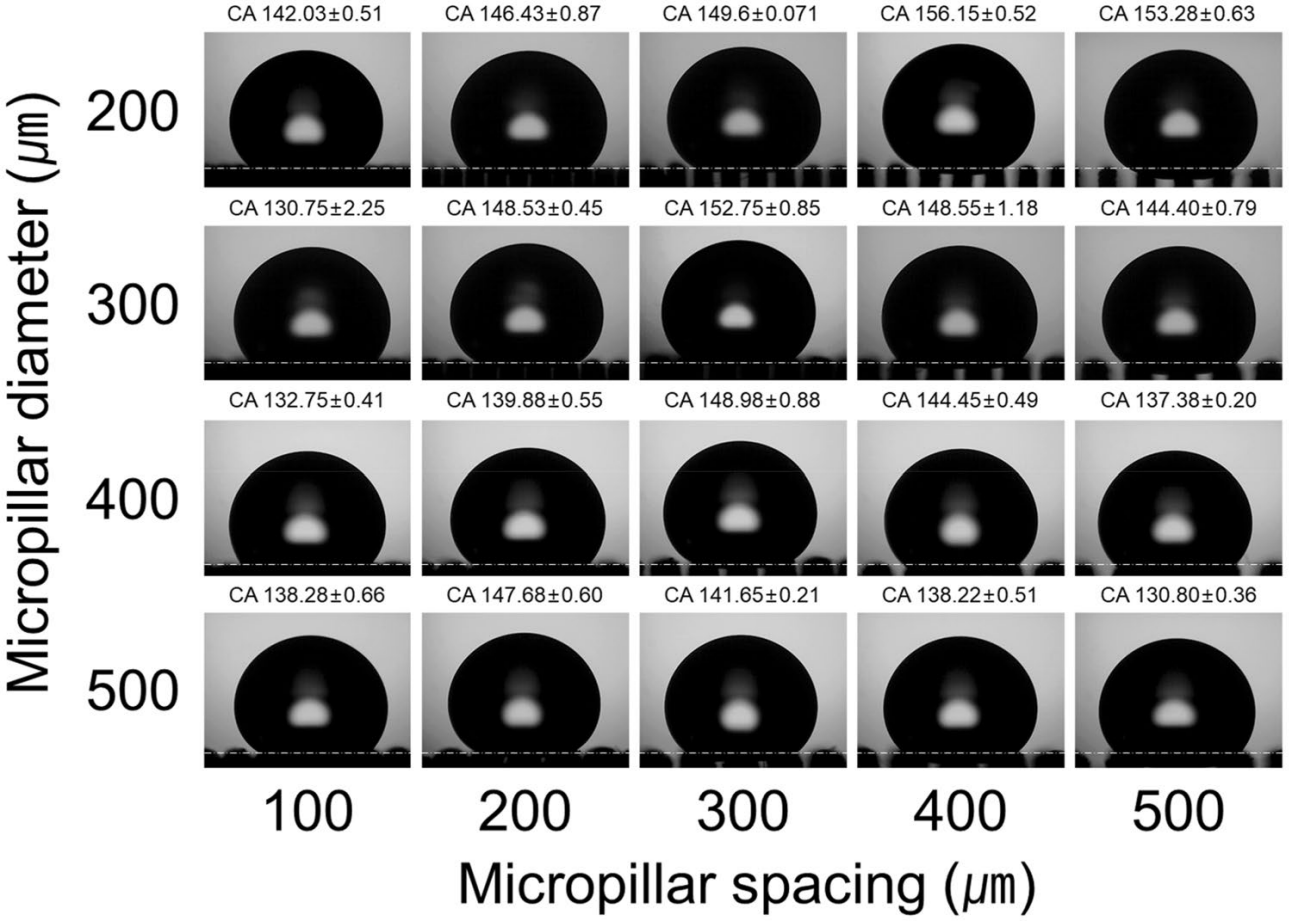
Images of the contact angle according to the diameter and spacing of micropillars.

When the contact angle is greater than that indicated by the red line in the figure, the water droplets are placed on the spherical surface. If the contact angle falls between the red and blue lines, the water droplets are placed on the torus structure. Values lower than the blue line indicate that the droplets have penetrated the filler column. This model was verified using experimental data, as shown in Fig. 14.

**Figure 15:**
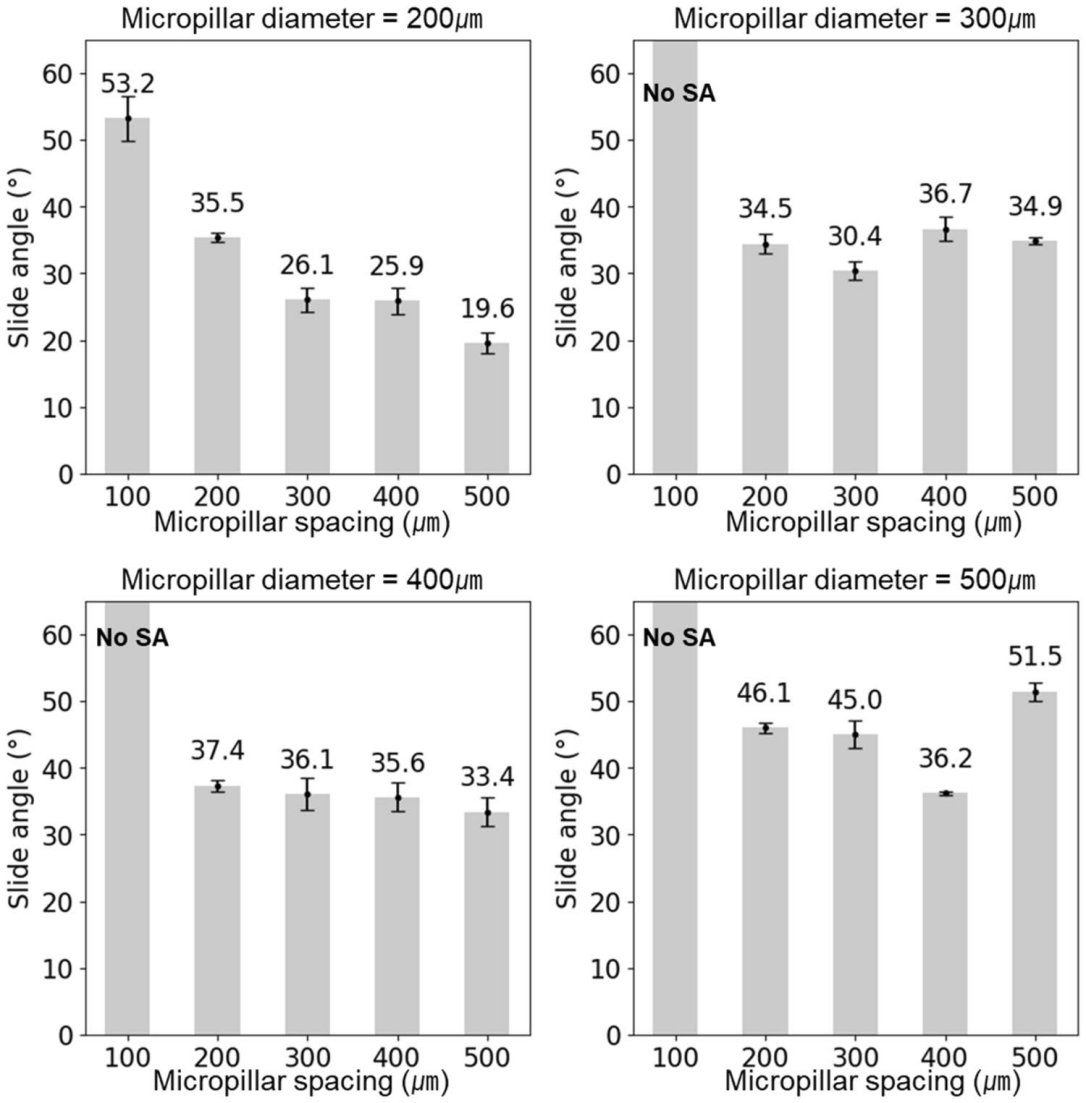
Sliding angle measurements according to diameter and spacing of micropillars.

Furthermore, the sliding angle, gauged by tilting a micropillar at 1.6 degrees/s with a 10 μL water droplet until its motion initiation, predominantly showed a declining trend with increasing micropillar spacing as shown in Fig. 15. The smallest sliding angle was particularly observed for the 200 µm diameter. This was attributable to the decreasing liquid–solid contact area, which intensified liquid–solid interactions^36^.

The sliding angle results revealed that, owing to the variable adhesion to water, the entire range exhibited a sliding angle from over 10° to no sliding angle. This can be considered as being controllable wettability from 20° of sliding angle to no sliding angle, which is the “petal effect” or “pinned effect” state^37^. It was inferred that the top portion of the micropillars produced using PBF assumed a spherical form, leading to the inevitable formation of an area where water can penetrate.

## Conclusion

In this study, the fabrication of a structure with controllable wettability using PBF of CP-Ti was comprehensively examined. A particular focus was the significance of specific micropillars fabricated by PBF in affecting wettability. The following conclusions were drawn from this study:

- To achieve a blend of high relative density, minimal porosity, and precision in micropillar production, the optimal PBF conditions were identified as a laser power of 85 W, a scan speed of 226 mm/s, and a volumetric energy density of 125 J/mm^3^.
- The wettability of the resulting structures was profoundly influenced by the micropillar diameter and spacing. Remarkable hydrophobicity with contact angles exceeding 150° was achieved under specific configurations, validating the efficacy of the methodology.
- Microscopic investigations revealed a consistent morphological trait: the micropillars exhibited a spherical top surface with adhered unmelted powder on their side. The study also proposed a simplified model, accounting for the challenging nature of defining precise contact areas owing to the presence of irregularly attached unmelted powder.
- By employing the Cassie–Baxter model according to the pillar morphology, insights into the water droplet interaction and penetration depth on the micropillar surfaces were obtained. This model differentiates between droplets residing on the spherical top and those interacting with the torus structure formed by the adhered powder.
- The “petal” or “pinned” effect, characterized by a contact angle exceeding 150° and a sliding angle greater than 10°, emerged owing to the distinct morphology of the micropillars, underscoring the critical role of micro-structural design in surfaces with controllable wettability.

In summary, this study successfully demonstrated the potential of PBF for fabricating surfaces with controllable wettability by harnessing the design of micropillars. The findings not only advance our understanding of the capabilities of PBF but also pave the way for innovations in the design and manufacture of water-repellent surfaces for various applications.

## Data Availability

The datasets used and/or analysed during the current study available from the corresponding author on reasonable request.

## References

[1] Song JW, Fan LW (2021). Temperature dependence of the contact angle of water: A review of research progress, theoretical understanding, and implications for boiling heat transfer. Adv. Colloid Interface Sci..

[2] Marmur A, Kojevnikova S (2020). Super-hydrophobic surfaces: Methodological considerations for physical design. J. Colloid Interface Sci..

[3] Wong TS, Sun T, Feng L, Aizenberg J (2013). Interfacial materials with special wettability. MRS Bull..

[4] Zhang C (2023). Shelter forest inspired superhydrophobic flame-retardant composite with root–soil interlocked micro/nanostructure enhanced mechanical, physical, and chemical durability. Adv. Funct. Mater..

[5] Xie H (2022). Cost-effective fabrication of micro-nanostructured superhydrophobic polyethylene/graphene foam with self-floating, optical trapping, acid-/alkali resistance for efficient photothermal deicing and interfacial evaporation. Small.

[6] Nyankson E, Agbe H, Takyi GKS, Bensah YD, Sarkar DK (2022). Recent advances in nanostructured superhydrophobic surfaces: Fabrication and long-term durability challenges. Cur. Opin. Chem. Eng..

[7] Park H, Choi CH, Kim CJ (2021). Superhydrophobic drag reduction in turbulent flows: A critical review. Exp. Fluids.

[8] Lambley H (2023). Freezing-induced wetting transitions on superhydrophobic surfaces. Nat. Phys..

[9] Sun X, Xue B, Tian Y, Qin S, Xie L (2018). 3D porous poly(l-lactic acid) materials with controllable multi-scale microstructures and their potential application in oil–water separation. Appl. Surf. Sci..

[10] Mohamed AMA, Abdullah AM, Younan NA (2015). Corrosion behavior of superhydrophobic surfaces: A review. Arab. J. Chem..

[11] Niu C, Liu Y, Shang D, Qin Q, Liu W (2022). Hydrodynamic noise reduction mechanism of a superhydrophobic surface with different slip velocities. J. Sound Vib..

[12] Zhang X, Wang L, Levänen E (2013). Superhydrophobic surfaces for the reduction of bacterial adhesion. RSC Adv..

[13] Kim W (2018). Engineering lotus leaf-inspired micro- and nanostructures for the manipulation of functional engineering platforms. J. Ind. Eng. Chem..

[14] Zaman Khan M (2022). Recent advances in superhydrophobic surfaces for practical applications: A review. Eur. Polym. J..

[15] Wu T (2022). Bioinspired micro/nanostructured polyethylene/poly(ethylene oxide)/graphene films with robust superhydrophobicity and excellent antireflectivity for solar-thermal power generation, thermal management, and afterheat utilization. ACS Nano.

[16] Alimohammadian M, Azizian S, Sohrabi B (2023). Preparation of the graphene-based smart hydrophobic nanocomposite and its application in oil/water separation. Sci. Rep..

[17] Xie H (2023). Efficient fabrication of micro/nanostructured polyethylene/carbon nanotubes foam with robust superhydrophobicity, excellent photothermality, and sufficient adaptability for all-weather freshwater harvesting. Small.

[18] Ijaola AO (2020). Wettability transition for laser textured surfaces: A comprehensive review. Surf. Interfaces.

[19] Yu M (2023). Laser interference additive manufacturing ordered Cu microstructure. Appl. Surf. Sci..

[20] Wang Q (2021). Switchable wettability control of titanium via facile nanosecond laser-based surface texturing. Surf. Interfaces.

[21] Wang D (2020). Design of robust superhydrophobic surfaces. Nature.

[22] Wu H (2019). Large area metal micro-/nano-groove arrays with both structural color and anisotropic wetting fabricated by one-step focused laser interference lithography. Nanoscale.

[23] Jiao L (2018). Femtosecond laser produced hydrophobic hierarchical structures on additive manufacturing parts. Nanomaterials.

[24] Chun DM, Ngo CV, Lee KM (2016). Fast fabrication of superhydrophobic metallic surface using nanosecond laser texturing and low-temperature annealing. CIRP Ann..

[25] Jafari R, Cloutier C, Allahdini A, Momen G (2019). Recent progress and challenges with 3D printing of patterned hydrophobic and superhydrophobic surfaces. Int. J. Adv. Manuf. Technol..

[26] Yang Y (2018). Recent progress in biomimetic additive manufacturing technology: From materials to functional structures. Adv. Mater..

[27] Davoudinejad A, Cai Y, Pedersen DB, Luo X, Tosello G (2019). Fabrication of micro-structured surfaces by additive manufacturing, with simulation of dynamic contact angle. Mater. Des..

[28] Dong Z (2021). 3D printing of superhydrophobic objects with bulk nanostructure. Adv. Mater..

[29] Dwivedi S, Dixit AR, Das AK, Nag A (2023). A novel additive texturing of stainless steel 316L through binder jetting additive manufacturing. Int. J. Precis. Eng. Manuf. Green Technol..

[30] Mekhiel S, Koshy P, Elbestawi MA (2021). Additive texturing of metallic surfaces for wetting control. Addit. Manuf..

[31] Sun J (2020). Study on selective laser melting 316L stainless steel parts with superhydrophobic surface. Appl. Surf. Sci..

[32] Wang X, Liu J, He Y, Wang Y (2018). Selective laser melting of ink-printed (SLM-IP) copper (Cu) nanoparticles (NPs) for facile controllable fabrication of super-hydrophobic surface. Surf. Coat. Technol..

[33] Sun X (2022). A biomimetic hierarchical structure on selective laser melting titanium with enhanced hydrophilic/hydrophobic surface. J. Alloys Compd..

[34] Kim D-H (2022). Fabrication of Superhydrophobic Metal Surface by Selective Laser Melting 3D Printing and Non-toxic Post-process.

[35] Milne AJB, Amirfazli A (2012). The Cassie equation: How it is meant to be used. Adv. Colloid Interface Sci..

[36] Rohrs C, Azimi A, He P (2019). Wetting on micropatterned surfaces: Partial penetration in the Cassie state and Wenzel deviation theoretically explained. Langmuir.

[37] Feng L (2008). Petal effect: A superhydrophobic state with high adhesive force. Langmuir.

